# Comparative lipidomics and volatile profiling reveal distinct aroma signatures in Arbas cashmere goat meat from grazing and housed feeding systems

**DOI:** 10.1186/s40104-026-01423-w

**Published:** 2026-06-22

**Authors:** Chengrui Zhang, Xin Du, Dingkun Fan, Jixian Zhang, Aoyu Wang, Zhihao Song, Pei Zhong, Rong Bai, Qiyu Diao, Naifeng Zhang

**Affiliations:** Key Laboratory of Feed Biotechnology of Ministry of Agriculture and Rural Affairs, Institute of Feed Research of Chinese Academy of Agricultural Sciences, Beijing, 100081 China

**Keywords:** Aroma profiles, Feeding pattern, Foodomics, Intramuscular fat, Lipid metabolism, Meat flavor

## Abstract

**Background:**

Feeding patterns significantly influence goat meat aroma profiles, which are closely related to muscle lipid composition. This relationship is especially significant for indigenous breeds like the Arbas cashmere goat, whose distinctive meat aroma has been traditionally recognized. Despite the known correlation between lipid metabolism and aroma formation, the specific mechanisms by which grazing vs. housed feeding systems modulate these processes in indigenous goat breeds remain poorly characterized. This study employed a comparative approach using integrated foodomics and lipidomics to investigate aroma profiles and lipid compositions in the *longissimus thoracis et lumborum* (*LTL*) muscle of Arbas cashmere goats raised under contrasting grazing and housed feeding systems.

**Results:**

The volatile profile suggests that fruity and milky notes primarily characterized the aroma of the *LTL* muscle in Arbas cashmere goats. However, notable differences were observed between grazing and housed feeding goats in terms of their aroma profiles. (Z)-4-Heptenal, ethyl hexanoate, ethyl decanoate, ethyl octanoate, ethyl acetate and ethyl 3-methylbutyrate were identified as key contributors to meat aroma. Differences in intramuscular fat, triglycerides, and phospholipid content were also detected between groups. Lipidomics analysis identified 1,465 lipid species, among which PC (15:0/22:6) was uniquely involved in glycerophospholipid metabolism, arachidonic acid metabolism and α-linolenic acid metabolism pathways, suggesting a central role in the synthesis of aroma-related compounds.

**Conclusions:**

These findings demonstrate that feeding systems modulate meat flavor through lipid metabolism pathways, particularly via differential accumulation of key aroma volatiles and targeted alterations in glycerophospholipid metabolism, providing a scientific basis for optimizing housed feeding strategies to enhance Arbas cashmere goat meat quality.

**Graphical Abstract:**

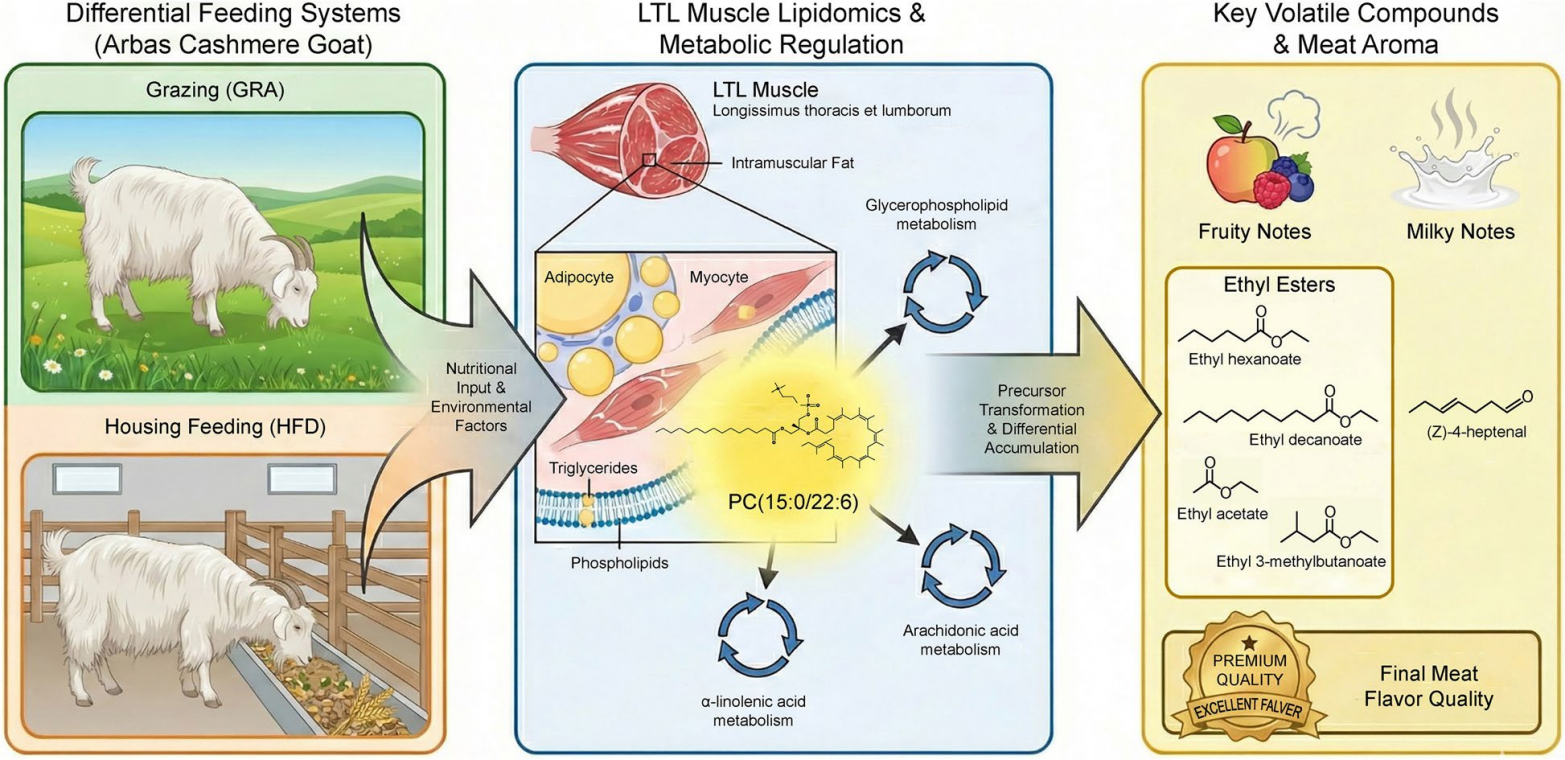

**Supplementary Information:**

The online version contains supplementary material available at 10.1186/s40104-026-01423-w.

## Background

The quality of premium goat meat encompasses not only superior sensory attributes and nutritional value but also emphasizes flavor as a critical determinant of consumer acceptance. Among the factors influencing flavor, volatile organic compounds (VOCs) play a decisive role in defining the unique aroma of meat products. Previous studies have identified aldehydes, ketones, phenolic compounds, and branched-chain fatty acids as key contributors to the characteristic aroma of meat [1]. The formation of these volatiles involves complex biochemical reactions, including Maillard reactions, thiamine degradation, and lipid oxidation, with lipid-derived compounds serving as major precursors of distinctive meat aromas [2]. Consequently, understanding the metabolic pathways and molecular targets involved in lipid oxidation is essential for elucidating the mechanisms underlying meat flavor formation.

Lipid composition and oxidative stability are considered central to the generation of aroma-active compounds during meat processing [3]. Lipid oxidation produces key secondary metabolites such as aldehydes, alcohols, ketones, and hydroxyepoxides through the α- and β-scission of peroxyl and alkoxyl radicals [4]. Among these, aldehydes and alcohols are recognized as the primary contributors to the characteristic aroma of mutton and chevon [5]. The oxidative susceptibility of polyunsaturated fatty acids (PUFAs), particularly linoleic acid (C18:2n-6) and α-linolenic acid (C18:3n-3), promotes the formation of critical flavor compounds such as hexanal and (E)-2-nonenal through radical-mediated pathways [6]. Meanwhile, medium-chain fatty acids (C10:0–C14:0) generate methyl ketones through β-oxidation, imparting roasted or creamy notes [7]. Furthermore, phospholipids, which are abundant in muscle cell membranes, have been reported to significantly contribute to aroma development by acting as essential precursors of VOCs [8]. These findings highlight the need to explore the biosynthetic pathways and transformation mechanisms of lipid-derived flavor compounds in detail.

Considering the significant impact of feeding systems on meat flavor and the key role of lipid oxidation in flavor formation, it is essential to investigate how different feeding regimes influence lipid composition and subsequent aroma development in Arbas cashmere goat meat. The meat of Arbas cashmere goats, a geographically protected and premium Chinese livestock resource, is well known for its rich nutritional value, unique sensory profile, and characteristic flavor compounds. However, traditional grazing systems have been increasingly challenged by overgrazing, pasture degradation, and extreme climatic events, leading to a shift toward intensive housed feeding systems through ecological restoration initiatives [9]. Feeding systems are known to influence meat flavor by modulating the metabolic pathways of amino acids, fatty acids, and other key precursors [10]. Grazing diets, particularly fresh pasture, are rich in plant-derived unsaturated fatty acids, which enhance the formation of specific intramuscular fatty acid profiles and contribute to the development of flavor precursor pools [11]. Recent studies have also demonstrated that grazing can regulate lipid oxidation pathways by influencing the activity of key enzymes in glycolipid metabolism, thereby altering the composition and ratios of volatile aroma compounds [3]. Nevertheless, the mechanistic links between feeding systems, lipid composition, and flavor formation, particularly the role of key lipid molecules in regulating characteristic aroma compounds remain poorly understood.

Given the knowledge gaps in the relationship between feeding systems, lipid composition, and flavor formation in Arbas cashmere goat meat, this study aimed to systematically elucidate the relationship between lipid composition and aroma formation in the samples under different feeding systems. By integrating multi-omics approaches, including GC–MS-based VOCs profiling and LC–MS-based lipidomics, along with histological analysis, we sought to identify the critical metabolic pathways and lipid-derived precursors driving aroma development. This research provides new insights into the lipid–aroma interaction network in goat meat and offers theoretical support for optimizing feeding strategies to enhance the flavor quality of Arbas cashmere goat meat under intensive farming systems.

## Materials and methods

### Experimental site and sampling

A total of 60 21-month-old Arbas cashmere goats (33.10 ± 2.44 kg) exhibiting good body condition were selected for this study. The goats were randomly divided into two treatments: grazing group (GRA, *n* = 30) and housed feeding group (HFD, *n* = 30). All goats were fed and watered ad libitum throughout the experimental period until 24 months of age (typical age of market readiness). To characterize the natural pasture, samples were collected in October and December 2024 to determine their botanical composition and chemical profile. Five 1 m × 1 m quadrats were established within the designated natural grazing area, spaced at intervals greater than 300 m. All herbaceous vegetation within each quadrat was harvested at ground level, manually sorted, and oven-dried at 65 °C for 48 h. The dried biomass was weighed to calculate botanical composition, then ground through a 1-mm sieve and stored for nutritional analysis. Goats in the grazing group were raised on the Otog desertified grassland (Inner Mongolia, China), a representative arid steppe whose unique botanical composition of drought-tolerant species serves as a critical environmental template for shaping the distinct metabolic profile and flavor of Arbas cashmere goat meat. The pasture's botanical composition was dominated by Asteraceae (approx. 40%; predominantly *Artemisia frigida* and *Artemisia ordosica*) and Poaceae (approx. 35%; mainly *Stipa breviflora* and *Stipa capillata*), followed by Fabaceae (approx. 10%; *Caragana spinifera* and *Sophora L.*) and other families (approx. 15%; including *Allium *spp. and *Convolvulus ammannii*). The nutrient composition of both experimental diets and the specific ingredients of the HFD are summarized in Table S1. At the end of the experimental period, 12 goats (*n* = 6 from per treatment) were randomly slaughtered. *Longissimus thoracis et lumborum* (*LTL*) muscle samples were then collected specifically for analyses. Goats were fasted for 24 h with free access to water until 2 h before the procedure. Subsequently, the goats were humanely euthanized via exsanguination, in strict accordance with veterinary regulatory protocols. After slaughter, goat carcasses were dry-aged at 0–4 °C for 24 h. Then, approximately 100 g of muscle tissue samples were collected from the *LTL* muscle of each goat. These samples were promptly placed in cryotubes and rapidly frozen in liquid nitrogen. They were then transported to the laboratory and stored in a deep freezer at −80 °C. This ensured the preservation of the samples for subsequent analysis of aroma characteristics, volatile metabolites, and lipid metabolomics.

### Determination of lipid metabolites

After 24 h of slaughtering of Arbas cashmere goats, the intramuscular fat (IMF) content in *LTL* was determined by Soxhlet extraction according to the method described in the Chinese national standard *National standard for food safety: determination of fat in food* (GB 5009.6–2016) [12]. All meat samples were taken and weighed at 0.5 g after freeze-drying and baked to complete dryness in a desiccator. IMF was separated in a Soxhlet extractor using anhydrous ether, and the samples were removed, vacuum-dried, and constant-weighted after 8 h. The IMF content was calculated by the following formula: IMF% = [weight before drying (g) − weight after drying (g)]/[weight before drying (g)] × 100%. According to the instructions for use of commercial kits (Beijing Sinouk Industrial Co., Ltd., Beijing, China), *LTL* triglyceride (TG), total cholesterol (TC), and phospholipid (PLIP) were measured using the colorimetric method.

### Electronic nose analysis

Ten grams of muscle tissue were accurately weighed and homogenized for 2 min to a mince-like consistency. Subsequently, portions of 5 ± 1 g of the homogenized meat were transferred into 50 mL headspace vials, which were immediately sealed with stoppers and crimp caps, and then left to equilibrate for 30 min at room temperature. In preparation for analysis, the PEN3 electronic nose system (Winmuster Airsense Analytics Inc., Schwerin, Germany) was powered on and allowed to warm up for 30 min to ensure stable baseline readings. Once the baseline was stabilized to unity, the measurement parameters were carefully configured as follows: a zero time of 15 s, a sample preparation time of 5 s, a sample measurement time of 280 s, and a cleaning time of 80 s. Both the injection flow rate and carrier gas flow rate were precisely set at 200 mL/min to maintain consistent sampling conditions. Following completion of the measurement, the electronic nose software was employed to conduct comprehensive data analysis, including loading plots, principal component analysis (PCA), and linear discriminant analysis (LDA), to extract relevant volatile compound information and assess sample differentiation.

### Volatile organic compounds analysis

A sample was taken into the 20 mL headspace bottle, and 10 μL of 2-octanol (10 mg/L stock in dH_2_O) as an internal standard was added. All samples were analyzed by Headspace Solid-Phase Microextraction-Gas Chromatography-Mass Spectrometry (HS–SPME–GC–MS). Automated SPME was performed using a PAL robotic autosampler (CTC Analytics AG, Zwingen, Switzerland), the incubation temperature is set to 60 °C, with a preheat time of 15 min, followed by a 30 min incubation period and a 4 min desorption phase. HS–SPME–GC–MS analysis was performed using an Agilent 7890 gas chromatograph system coupled with a 5977B mass spectrometer. The system utilized a DB-Wax column with injection in splitless mode. Helium was used as the carrier gas, the front inlet purge flow was 3 mL/min, and the gas flow rate through the column was 1 mL/min. The initial temperature was kept at 40 °C for 4 min, then raised to 245 °C at a rate of 5 °C/min, and kept for 5 min. The injection, transfer line, ion source and quad temperatures were 250, 250, 230, and 150 °C, respectively. The energy was −70 eV in electron impact mode. The mass spectrometry data were acquired in scan mode with the *m*/*z* range of 20–400, solvent delay of 2.37 min.

Data processing, including raw peak extraction, baseline filtering, alignment, deconvolution, and peak identification, was performed using Chroma TOF 4.3X software (LECO Corporation). Metabolites were identified by matching mass spectra and retention indices against the NIST and Fiehn libraries [13].

### Relative odor activity value analysis

Relative odor activity value (ROAV) is employed to quantitatively assess each volatile compound's contribution to the test sample's overall flavor, spotlighting the key flavor compounds in the process. In different treatments, we calculated the detection rate of compounds, retaining those with a rate exceeding 50% and a variation coefficient below 30%. Subsequently, we determined the relative percentage (C%) of the average peak area for the retained compounds in each group. Compounds annotated with flavor sensory thresholds were then preserved. In this context, the ROAV value (ROAV stan) of the compound contributing most significantly to the sample group's overall flavor was defined as 100. Following this, the ROAV values (ROAV_A_) of the remaining compounds were calculated.

$${ROAV}_{A}\approx 100\times \frac{{C\%}_{A}}{{C\%}_{stan}}\times \frac{{T}_{stan}}{{T}_{A}}$$

C%_A_: The relative percentage of the average peak area of compounds other than those contributing most significantly to the sample group's overall flavor.

C%_stan_: The relative percentage of the average peak area of the compound contributes most to the sample group's overall flavor.

T_stan_: The sensory threshold of the compound contributing most to the sample group's overall flavor.

T_A_: The sensory threshold of other compounds.

Ordinarily, ROAV ≥ 1 were the key flavor compounds, while those with 0.1 ≤ ROAV < 1 were important modifiers of the overall flavor.

### Lipid extraction and data analysis

Lipid extraction was conducted by homogenizing 50 mg of *LTL* samples in 2 mL microtubes with 280 μL methanol/water (2:5, v/v) and 400 μL methyl tert-butyl ether (MTBE), supplemented with a single 6 mm grinding bead. Homogenization was performed at −10 °C using a Wonbio-96c high-throughput tissue grinder (Shanghai Wonbio Technology Co., Ltd.) at 50 Hz for 6 min, followed by ice-cooled ultrasonication (40 kHz, 30 min, 5 °C). After incubation at −20 °C for 30 min, samples were centrifuged (13,000 × *g*, 4 °C, 15 min), and 350 μL of the upper lipid phase was collected, dried under nitrogen gas, and reconstituted in 100 μL isopropanol/acetonitrile (1:1, v/v) with brief 5 °C water bath sonication. Before UHPLC-MS/MS analysis, samples were centrifuged again (13,000 × *g*, 4 °C, 15 min), and 2 μL of supernatant was injected into the system.

The LC–MS/MS analysis was performed on a Thermo Q Exactive HF-X system (Majorbio Bio-Pharm, Shanghai, China) with an Accucore C30 column (100 mm × 2.1 mm, 2.6 μm; Thermo, USA). Mobile phases: (A) 10 mmol/L NH_4_Ac in ACN/H_2_O (1:1, 0.1% FA); (B) 2 mmol/L NH_4_Ac in ACN/IPA/H_2_O (10:88:2, 0.02% FA). Gradient elution at 0.4 mL/min (2 μL injection, 20 min runtime; 40 °C), all of samples maintained at 4 °C during the period of analysis. MS detection used a HESI source (± 3.0 kV) with sheath/auxiliary gas 60/20 psi (370 °C). DDA acquisition (200–2,000 *m/z*) employed stepped NCE (20 eV/40 eV/60 eV). After UPLC-MS/MS analyses, the raw data were imported into Lipid Search (Thermo, CA, USA) for peak detection, alignment, and identification. The data were analyzed through the free online platform of the Majorbio cloud platform (cloud.majorbio.com).

Differential metabolites were identified by combining variable importance in projection (VIP) > 2 [14] from OPLS-DA and *t*-test, *P* < 0.05. Pathway enrichment analysis of selected metabolites was performed through Kyoto Encyclopedia of Genes and Genomes (KEGG) mapping, with significant pathways (Fisher's exact test, *P* < 0.05) classified by biological functions using scipy.stats (Python packages) (https://docs.scipy.org/doc/scipy/).

### Statistical analysis

IMF, TG, TC, and PLIP results were presented as mean ± SEM following triplicate determinations. Based on the assessment of data variance, an independent samples *t*-test was used to evaluate the differences between the GRA and HFD group, employing a statistical significance threshold of ^*^P < 0.05, ^**^P < 0.01, and ^***^P < 0.001. All analyses were performed using the vegan R package (Version 4.5.0). Identify lipid metabolites of significance based on variables that meet the VIP > 2 criterion. Spearman's correlation analysis was performed to evaluate the relationships among the aroma traits of goat meat, VOCs, and the lipid profile.

## Results

### Aroma profile differences in the *LTL* muscle of Arbas cashmere goats under different feeding systems

**Fig. 1:**
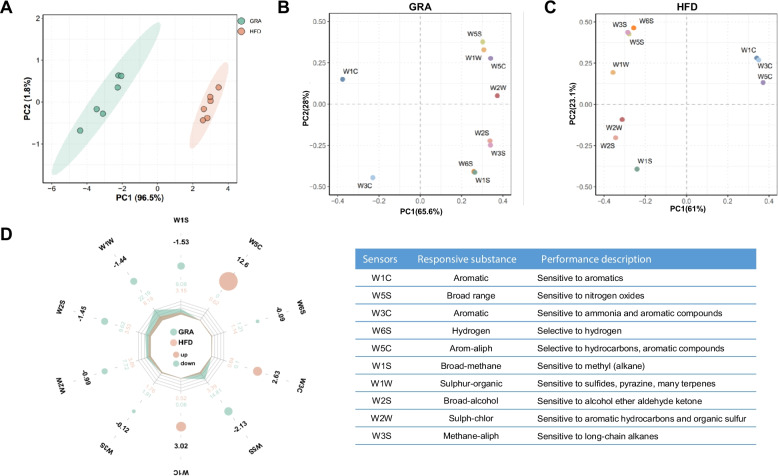
Aroma profiling of *LTL* under different feeding systems. **A** PCA score plot of E-nose data showing separation between GRA and HFD. **B** and **C** PCA loading plots for GRA and HFD, respectively. **D** E-nose sensor responses and radar map

In this experiment, electronic nose analysis was performed on samples of the *LTL* muscle from Arbas cashmere goats raised under two feeding systems: HFD and GRA, to compare the differences in their aroma profiles. As shown in Fig. 1A, a significant difference in the aroma composition of the *LTL* muscle was observed between the two feeding systems. The PCA loading diagram revealed that the primary odor compounds contributing to the first principal component of the *LTL* muscle in Arbas cashmere goats from the GRA group were W5C (aromatic and olefinic polar molecules), W2W (aromatic and organosulfur compounds), and W3S (long-chain alkanes), with their contributions ranked as W2W > W5C > W3S. For the second principal component, the contributing odor compounds were W5S (oxygen–nitrogen compounds), W1W (sulfur compounds), and W2W, with the contribution magnitude in the order of W5S > W1W > W2W (Fig. 1B). In the HFD group, the primary contributing odor compounds to the first principal component were W1C (aromatic compounds), W3C (ammonia and aromatic compounds), and W5C (aromatic and olefinic polar molecules), with their contribution magnitude in the order of W5C > W3C > W1C. For the second principal component, the contributing odor compounds included W5S (oxygen–nitrogen compounds), W3S (long-chain alkanes), and W6S (hydrides), with the contribution magnitude ranked as W6S > W3S > W5S (Fig. 1C). Furthermore, electronic nose analysis revealed significant differences in the aroma response levels of the *LTL* muscle under different feeding systems (Fig. 1D), with more pronounced changes observed in the aroma responses of W1W, W5S, W2S, and W1S (Table S2).

### Different feeding systems alter the VOCs of Arbas cashmere goat *LTL* muscle: esters driven aroma divergence

**Fig. 2:**
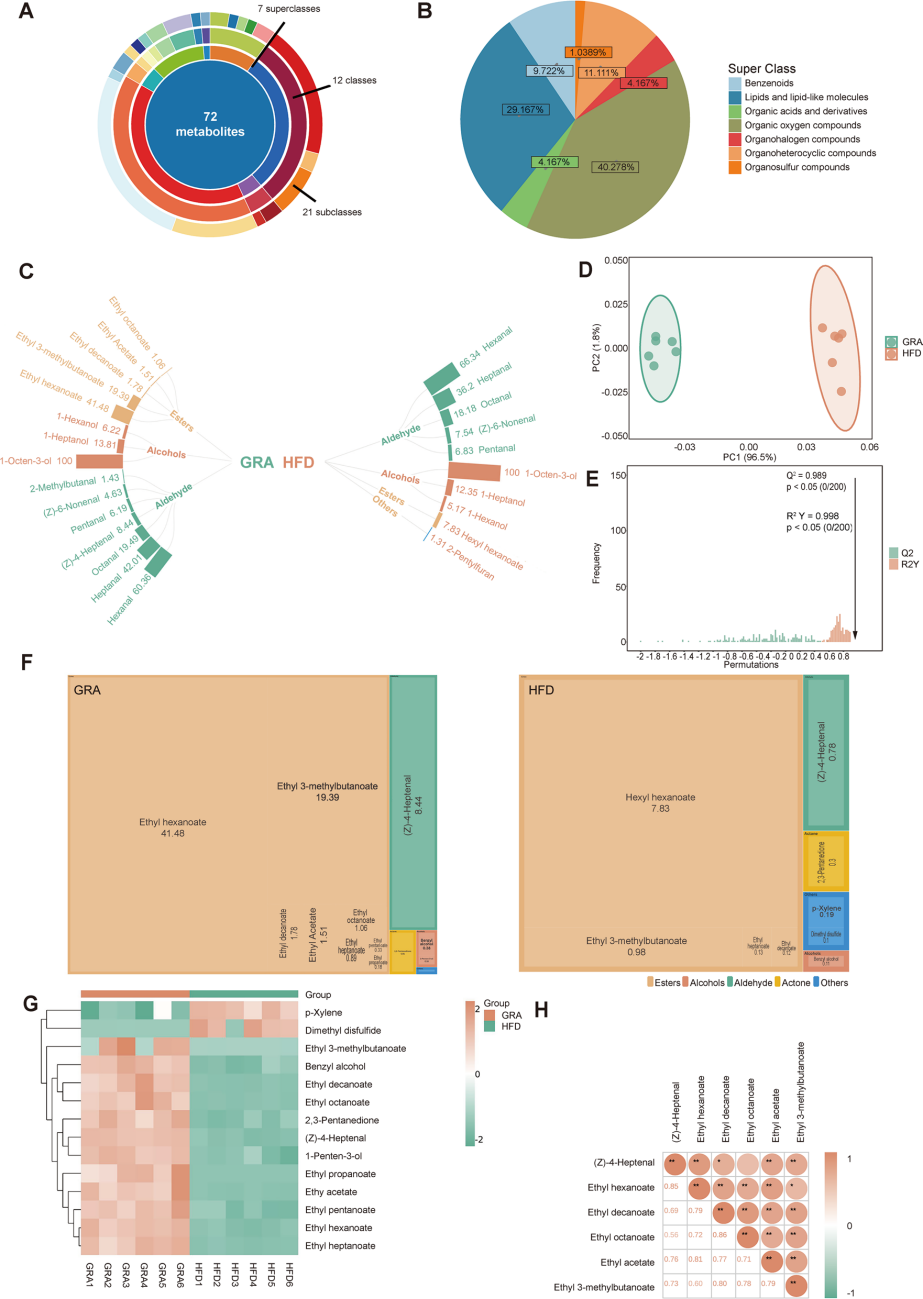
Volatile composition and key aroma contributors in *LTL. ***A** Number of volatiles detected. **B** Class distribution of volatiles. **C** Characteristic aroma compounds and their ROAV contributions. **D** and **E** PCA score plot and OPLS-DA permutation test for GRA vs. HFD (*R*^2^*Y* = 0.998, *Q*^2^ = 0.989). **F** Differences in characteristic volatiles with ROAV ≥ 0.1. **G** Heatmap of characteristic volatiles across feeding systems. **H** Correlations among key aroma compounds (ROAV ≥ 1); ^*^|*r*| > 0.6, ^**^|*r*| > 0.7

Volatile metabolites from the *LTL* muscle of Arbas cashmere goats were analyzed using HS-GC–MS under different feeding systems, revealing 72 flavor-active compounds (Fig. 2A). These compounds were categorized into seven main groups: Benzenoids, Lipids and lipid-like molecules, Organic acids and derivatives, Organic oxygen compounds, Organohalogen compounds, Organoheterocyclic compounds, and Organosulfur compounds (Fig. 2B). Specifically, the composition included 17 esters, 14 alcohols, 11 aldehydes, 8 ketones, 7 sulfur-containing compounds, 6 aromatics, 4 halogenated hydrocarbons, 3 acids, and 2 other compounds (Table S3). Notably, the HFD group exhibited significantly higher levels of benzene, ethylbenzene, m-xylene, p-xylene, butanoic acid, butyrolactone, and methyl hexanoate compared to the GRA group. In contrast, the GRA group had higher concentrations of hexanal, heptanal, octanal, 3-hexanol, and 3-heptanol (Fig. S1). To evaluate the contribution of individual aroma compounds to the overall aroma of goat meat, ROAV were calculated. A total of 15 key aroma compounds (ROAV ≥ 1) were identified in the *LTL* muscle of goats in the GRA group, mainly comprising aldehydes, alcohols, and esters. In contrast, 10 characteristic compounds were detected in the HFD group, including aldehydes, alcohols, esters, and a small proportion of furans (Fig. 2C). Among these, 1-octen-3-ol, hexanal, heptanal, ethyl hexanoate, ethyl 3-methylbutyrate, and 1-heptanol were consistently present in both groups and considered the core aroma compounds. Notably, quantitative analysis revealed that the concentrations of all these key volatiles were significantly higher in the GRA group compared to the HFD group (Table S3). These substances contributed primarily to fruity, grassy, mushroom, sweet, floral, buttery, and dairy (Table S4).

Principal component analysis (PCA) of the volatile metabolites in the *LTL* muscle under grazing and housed conditions showed significant differences in the volatile composition between the two feeding systems, with no significant variation within each group (Fig. 2D). The OPLS-DA permutation test further confirmed the robustness and reliability of the data (Fig. 2E). To further clarify the impact of different feeding systems on the aroma profile of the *LTL* muscle in Arbas cashmere goats, we conducted a comparative analysis of key aroma compounds. Comparative analysis revealed a predominantly decreasing trend in the HFD group, with 26 compounds showing reduced levels compared to only 13 compounds showing increased levels relative to the GRA group (Fig. S2A). Using the criteria of variable importance in projection (VIP) > 1, fold change (FC) > 2, or FC < 0.5, we identified 30 differential aroma compounds (Fig. S2B). In the GRA group, 13 characteristic aroma compounds (ROAV ≥ 0.1) were detected, including eight esters, two alcohols, one aldehyde, one ketone, and two miscellaneous compounds. In contrast, the HFD group had nine characteristic aroma compounds, consisting of four esters, one alcohol, one aldehyde, one ketone, and two miscellaneous compounds (Fig. 2F). Notably, levels of p-xylene and dimethyl disulfide significantly decreased in the HFD group, while the remaining characteristic aroma compounds significantly increased (Fig. 2G). Further analysis revealed six key aroma compounds (ROAV ≥ 1) in the GRA group: (Z)-4-heptenal, ethyl hexanoate, ethyl decanoate, ethyl octanoate, ethyl acetate, and ethyl 3-methylbutyrate. Ethyl hexanoate was the unique key aroma compound present in both groups, yet it exhibited a significantly higher concentration in the GRA group (Table S5). We summarized the contribution of six key aroma compounds to the flavor characteristics and identified their roles in defining specific sensory profiles. Among these, (Z)-4-heptenal was the primary contributor to the milky flavors associated with terms such as Cream, Milky, Dairy, Creamy, and Sweet. Ethyl hexanoate played a significant role in the fruity notes of pineapple, banana, fruit, and apple. Ethyl decanoate was found to contribute to the flavors of apple, grape, pea, and brandy, while ethyl octanoate was a major contributor to the aromas of apple, grape, pea, and brandy, as well as to those of banana, pineapple, pear, apricot and floral. Lastly, ethyl 3-methylbutyrate enhanced the fruity profiles, particularly those of apple, pineapple, grape and general fruity notes (Table S5). Correlation analysis indicated strong pairwise relationships (*r* > 0.7) between ethyl hexanoate and all other key aroma compounds except ethyl 3-methylbutyrate (Fig. 2H), suggesting that ethyl hexanoate plays a central role in modulating the aroma profile of Arbas cashmere goat meat. Finally, the lipid profile of the *LTL* muscle of Arbas cashmere goats was determined to further explore the lipid differences between the two feeding systems and to gain deeper insight into the lipidomics of the meat.

### Feeding systems alter IMF deposition and lipidome composition in *LTL* muscle of Arbas cashmere goats

**Fig. 3:**
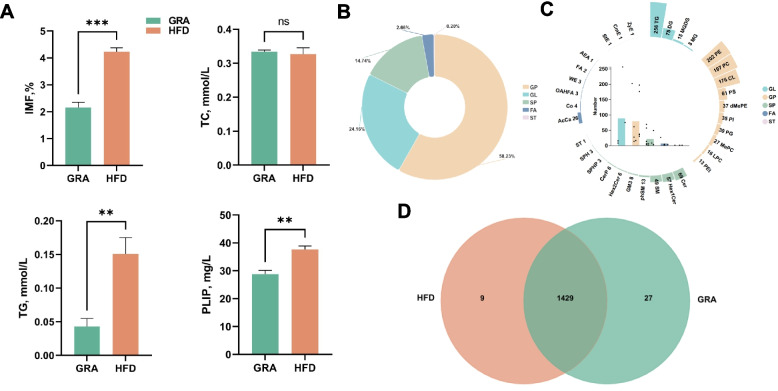
Lipid phenotype and composition of *LTL* under different feeding systems. **A** Intramuscular fat (IMF), total cholesterol (TC), triglycerides (TG), and phospholipids (PLIP). **B** Lipid class composition and proportions. **C** Lipid subclasses and counts. **D** Venn diagram of shared and unique lipid species between groups. ^ns^*P* > 0.05; ^*^P < 0.05; ^**^P < 0.01; ^***^P < 0.001

Significant differences were observed in the *LTL* muscles of Arbas cashmere goats under different feeding systems. The content of IMF, TG, and PLIP in the muscles of goats from the HFD group was significantly higher compared to those in the GRA group (*P* < 0.05). However, total cholesterol (TC) levels were not significantly affected by the feeding systems (*P* > 0.05) (Fig. 3A). Based on these findings, lipidomics analysis was conducted on the *LTL* muscles of Arbas cashmere goats under both feeding conditions. Qualitative analysis of the lipid composition using LC–MS in both positive and negative ion modes identified a total of 1,465 lipid molecules. These were classified into five major categories: 853 glycerophospholipids (GP, 58.23%), 354 glycerolipids (GL, 24.16%), 216 sphingolipids (SP, 14.74%), 39 fatty acids (FA, 2.66%), and 3 sterols (STs, 0.20%) (Fig. 3B). These lipids could be further divided into 33 subclasses, including 202 phosphatidylethanolamines (PE), 197 phosphatidylcholines (PC), 176 cardiolipins (CL), 61 phosphatidylserines (PS), 37 dimethylphosphatidylethanolamines (dMePE), 35 phosphoinositides (PI), 30 phosphatidylglycerols (PG), 27 methylphosphatidylcholines (MePC), 16 lysophosphatidylcholines (LPC), 13 phosphatidylethanolamines (PEt), 256 triglycerides (TG), 75 diglycerides (DG), 15 monogalactosyldiacylglycerols (MGDG), 8 monoglycerides (MG), 69 ceramides (Cer), 57 hexosylceramides (Hex1Cer), 49 sphingomyelins (SM), 13 phosphosphingomyelins (phSM), 38 glycosphingolipids (GM), 6 hexosylceramides (Hex2Cer), 6 ceramide phosphates (CerP), 3 sphingosine phospholipids (SPHP), 3 sphingoid bases (SPH), 1 steryl ester (ST), 26 acylcarnitines (AcCa), 4 coenzyme A derivatives (Co), 3 (O-acyl)-1-hydroxy fatty acids (OAHFA), 3 wax esters (WE), 2 fatty acids (FA), 1 anandamide (AEA), 1 steryl ester (StE), 1 ceramide methyl ester (CmE), and 1 zymosterol ester (ZyE) (Fig. 3C). Venn diagram analysis revealed that 1,429 lipid molecules were shared between the two feeding groups, while 9 species were exclusive to the HFD group and another 27 species were unique to the GRA group (Fig. 3D).

### PC (15:0/22:6) as a metabolic hub: changes in feeding systems modulated lipid metabolism network remodeling in Arbas cashmere goat muscle

**Fig. 4:**
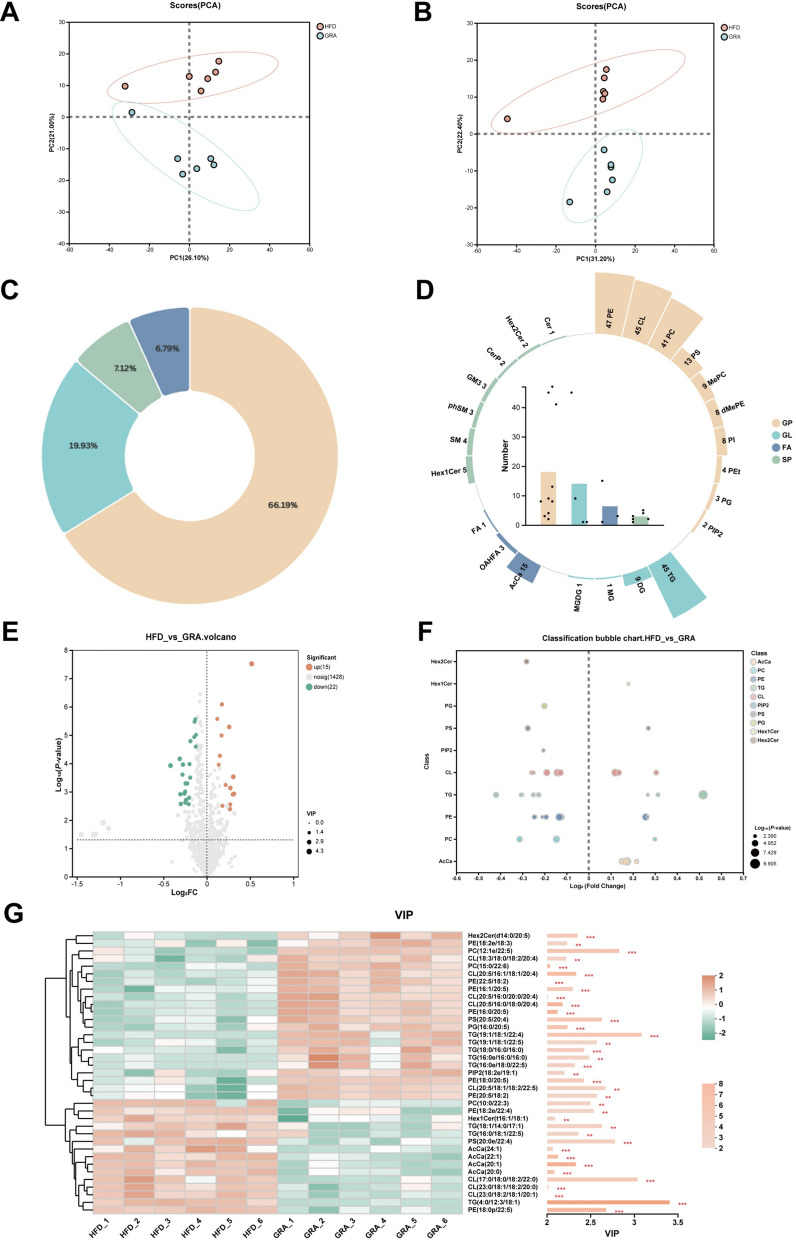
Differential lipidomics of *LTL*. **A** and **B** PCA score plots in positive and negative ion modes. **C** Class composition and proportions of differential lipids. **D** Subclass distribution and richness. **E** Volcano plot of significant lipids. **F** Subclass analysis of significant lipids. **G** VIP scores (screening: VIP > 2 and FC > 2 or FC < 0.5)

To investigate the expression of differential lipid molecules, we analyzed muscle samples from Arbas cashmere goats under different feeding systems using PCA and OPLS-DA. The samples from the HFD and GRA groups were dispersed in positive and negative ion modes, while samples within each group were aggregated, indicating significant differences in lipid composition between the two feeding systems (Fig. 4A and B). A total of 281 differential lipid molecules were identified, with glycerophospholipids (GP) accounting for 66.19%, including 47 PEs, 45 CLs, 41 PCs, 13 PSs, 9 MePCs, 8 dMePEs, 8 PIs, 4 PEts, 3 PGs, and 2 PIP2s. Glycerolipids (GL) made up 19.93%, consisting of 45 TGs, 9 DGs, 1 MG, and 1 MGDG. Sphingolipids represented 7.12%, including 5 Hex1Cer, 4 SM, 3 phSM, 3 GM3, 2 CerP, 2 Hex2Cer, and 1 Cer. Fatty acids (FA) accounted for 6.79%, containing 15 AcCa species, 3 OAHFA species, and 1 FA species (Fig. 4C and D). To identify key lipid molecules, we applied the criteria of VIP > 2, FC > 2, or FC < 0.5. This analysis revealed 15 lipids that were significantly up-regulated and 22 that were significantly down-regulated (Fig. 4E). A VIP heatmap was used to visualize the abundance of 37 differentially expressed lipid molecules in detail (Fig. 4G). We found that the HFD group had the highest abundance of up-regulated lipids, such as TG (4:0/12:3/18:1), AcCa (20:1), AcCa (22:1), and PE (18:0p/22:5), while the GRA group had the most abundant down-regulated lipids, including CL (20:5/16:0/18:0/20:4), PE (16:0/20:5), PC (15:0/22:6), and CL (20:5/16:1/18:1/20:4).

As shown in Fig. 4F, these differential lipid molecules were classified into 10 subclasses, including AcCa, PC, PE, TG, CL, PIP2, PS, PG, Hex1Cer, and Hex2Cer. Lipids such as TG, PE, AcCa, and PC were significantly differentiated between the two feeding systems. Specifically, Hex1Cer and AcCa lipid subclasses were predominantly enriched in the HFD group, while Hex2Cer and PG lipids were more abundant in the GRA group, indicating their role in muscle metabolism under different feeding systems. Further analysis categorized these differentially metabolized lipids based on their unsaturated bonds. We found that the levels of saturated fatty acids 10:0, 20:0, 22:0, and 4:0 were significantly higher in the HFD group, while the contents of saturated fatty acids 16:0 and 18:0 were significantly lower (*P* < 0.05) (Fig. S3A). Monounsaturated fatty acids 12:1, 16:1, and polyunsaturated fatty acids 18:1 were significantly reduced in the HFD group, whereas polyunsaturated fatty acids 18:3, 20:4, 20:5, 22:5, and 22:6 were also significantly reduced. On the other hand, fatty acids 20:1, 22:1, 24:1, and 22:3 showed significant increases in the HFD group (*P* < 0.05) (Fig. S3B and C).

**Fig. 5:**
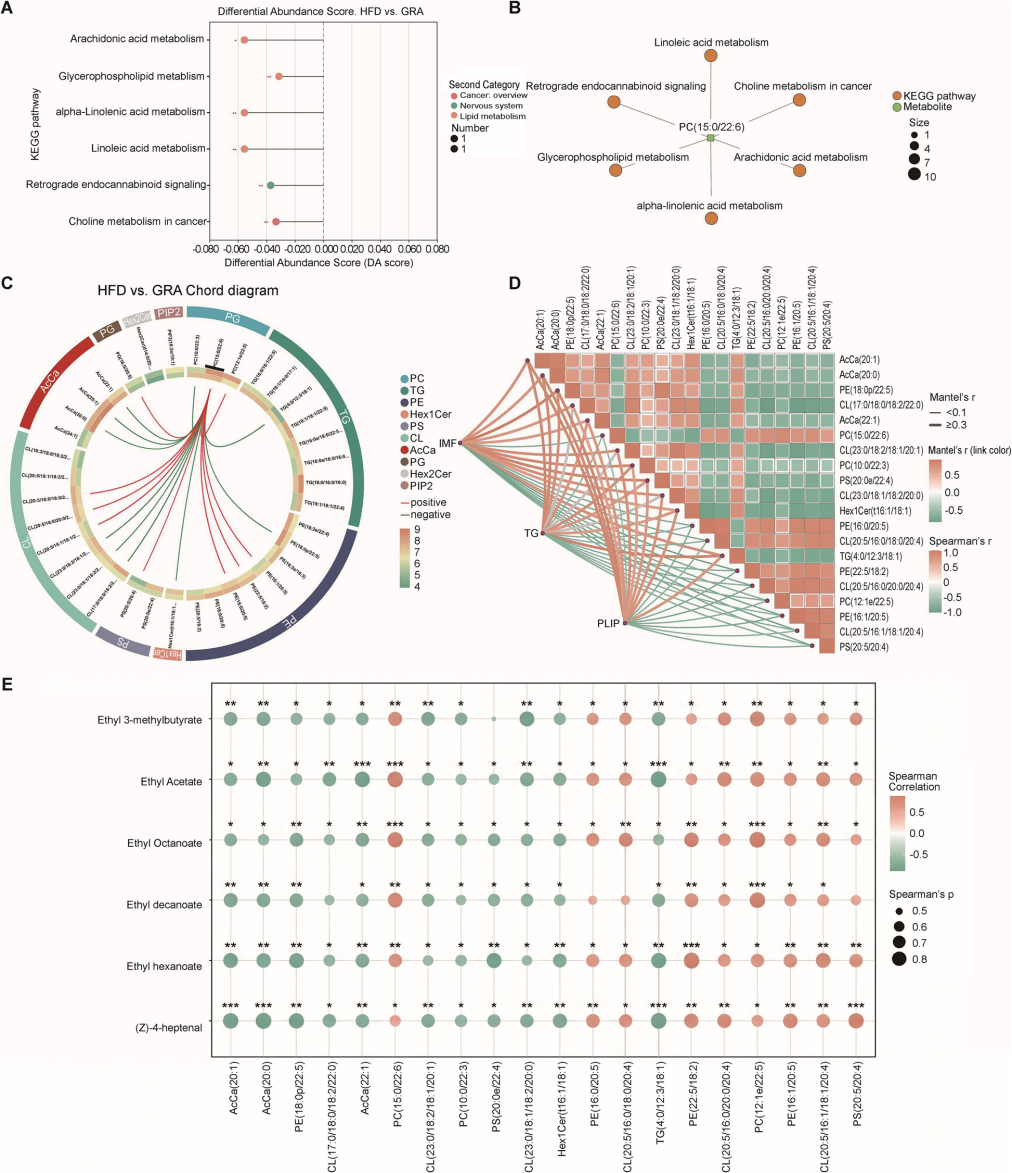
Pathway prediction and lipid-phenotype/aroma associations. **A** KEGG enrichment of differential lipids. **B** PC (15:0/22:6) identified as the only key lipid enriched across predicted pathways. **C** Correlation and chord diagram of PC (15:0/22:6) with other differential lipids. **D** Mantel test (Spearman) linking lipid phenotypes to key lipid molecules; orange, positive; green, negative. **E** Clustered bubble plot (Spearman) relating key aroma compounds to key lipids; orange, positive; green, negative. ^*^P < 0.05; ^**^P < 0.01; ^***^P < 0.001

To further explore the metabolic pathways associated with these differential lipid molecules, KEGG pathway enrichment analysis was performed. The results identified six major metabolic pathways enriched in the differential lipids (*P* < 0.05) (Fig. 5A). These pathways included glycerophospholipid metabolism, arachidonic acid metabolism, and alpha-linolenic acid metabolism, all of which are involved in lipid metabolism synthesis. Notably, PC (15:0/22:6) was found to participate in all three pathways related to lipid metabolism synthesis (Fig. 5B). Lastly, we analyzed the differential lipid molecules interacting with PC (15:0/22:6) and found significant positive correlations with lipids such as PG (16:0/20:5), PC (12:1e/22:5), PE (16:1/20:5), PE (22:5/18:2), PE (16:0/20:5), CL (20:5/16:0/18:0/20:4), CL (20:5/16:0/20:0/20:4), CL (20:5/16:1/18:1/20:4), and PS (20:5/20:4). Conversely, negative correlations were observed with lipids like AcCa (20:1), AcCa (20:0), PE (18:0p/22:5), CL (17:0/18:0/18:2/22:0), AcCa (22:1), CL (23:0/18:2/18:1/20:1), PC (10:0/22:3), CL (23:0/18:1/18:2/20:0), Hex1Cer (t16:1/18:1), and TG (4:0/12:3/18:1) (Fig. 5C).

### PC (15:0/22:6) as a key target linking *LTL* muscle lipid phenotypes and aroma compounds under different feeding systems

To identify potential predictors of changes in key lipid molecules in the muscles of Arbas cashmere goats under different feeding systems, we performed correlation analyses between their lipid phenotypes and 20 representative lipid metabolites (Fig. 5D). The results revealed that PC (15:0/22:6) was significantly negatively correlated with IMF, triglycerides (TG), and phospholipid-like molecules (PLIP). In contrast, TG (4:0/12:3/18:1), Hex1Cer (t16:1/18:1), PS (20:0e/22:4), PC (10:0/22:3), AcCa (22:1), AcCa (20:1), AcCa (20:0), and PE (18:0p/22:5) showed significant positive correlations with these lipid phenotypes (*P* < 0.05). Interestingly, these differential lipid molecules were strongly negatively correlated with PC (15:0/22:6) (*P* < 0.05). Since the metabolic activities of lipid molecules, which are essential for most volatile metabolites, are closely linked to aroma levels, we further correlated the six key aroma compounds identified earlier with the lipid molecules (Fig. 5E). We found that PC (15:0/22:6) exhibited strong positive correlations with the following six aroma compounds: (Z)-4-heptenal, ethyl hexanoate, ethyl decanoate, ethyl octanoate, ethyl acetate, and ethyl 3-methylbutyrate (*P* < 0.05). On the other hand, TG (4:0/12:3/18:1), Hex1Cer (t16:1/18:1), PS (20:0e/22:4), PC (10:0/22:3), AcCa (22:1), AcCa (20:1), AcCa (20:0), and PE (18:0p/22:5) lipids showed significant negative correlations with these six key aroma compounds (*P* < 0.05). This result is consistent with the observed variation in PC (15:0/22:6).

## Discussion

The aroma of goat meat has long played a key role in influencing consumer preferences, and the distinctive aroma of Arbas cashmere goat meat is a major factor contributing to its popularity [15]. However, the transition from traditional grazing to intensive housing systems may alter the nutritional intake and metabolic activity of goats, potentially affecting the development of meat aroma. To investigate how feeding systems influence the aroma profile of Arbas cashmere goat meat, we conducted a comparative analysis of the *LTL* muscles from goats raised under grazing and housed conditions. Using an electronic nose, which enables rapid and comprehensive assessment of food aroma characteristics [16], we observed higher sensor responses for W1W and W5S (associated with oxygen- and nitrogen-containing compounds), and W2S (associated with aldehydes, ketones, and alcohols) across both feeding systems. These results suggest that sulphur-containing compounds, oxy-nitrogen compounds, aldehydes, ketones, and alcohols are the predominant contributors to the overall aroma of Arbas cashmere goat meat. Notably, in the grazing group, the main contributors to the first and second principal components were organosulfur compounds (W2W) and oxy-nitrogen compounds (W5S), respectively. In contrast, for the housed group, aromatic and olefinic polar molecules (W5C) and hydrides (W6S) were the dominant contributors. These differences indicate that the feeding system influences the composition of aroma-active compounds in goat meat, resulting in distinct aroma characteristics between grazing and housed goats. However, the electronic nose effectively distinguished overall aroma systems; it lacked specificity in identifying individual aroma compounds. To further investigate the changes in aroma composition, HS–SPME–GC–MS was employed to analyze differential aroma compounds with aroma presentation.

The transition from grazing to housed feeding altered the characteristic aroma profile of Arbas cashmere goat meat, primarily due to changes in the composition and abundance of VOCs, as well as the complex interactions among them [17]. In the current study, we found that the aroma of the *LTL* muscle under both feeding systems was predominantly shaped by esters, aldehydes, and alcohols. Among these, 1-octen-3-ol emerged as the dominant contributor, exhibiting the highest ROAV of 100 in both the GRA and HFD groups (Table S5), thereby imparting mushroom-like, fatty, grassy, and fruity notes. Other major contributors included hexanal, heptanal, and ethyl hexanoate, which are associated with grassy, fruity, and milky aromas. In addition, compounds such as ethyl 3-methylbutyrate and 1-heptanol exhibited similar aroma profiles, further reinforcing the conclusion that the characteristic aroma of Arbas cashmere goat meat is defined by fresh and milky notes. Notably, the abundance of several key aroma compounds was significantly reduced in the housed feeding group, in line with electronic nose results, suggesting a decline in beneficial aroma compounds after the shift in feeding strategy. This trend is consistent with previous findings in other breeds, such as Sunit sheep [11]. VOCs with ROAV values between 0.1 and 1 are considered aroma modifiers, while those with values above 1 are recognized as primary contributors to meat aroma [18]. Based on this classification, further analysis revealed that the differences in VOCs between feeding modes mainly involved esters, aldehydes, alcohols, and ketones. Alcohols are generally derived from the oxidation of conjugated linoleic acid [19], ketones result from lipid oxidation and hydrolysis [20], and aldehydes and esters are primarily produced through fatty acid metabolism [21]. These findings confirm that lipid-derived precursors are a key source of meat aroma. From this analysis, six key aroma compounds (ROAV > 1) were identified as major contributors to the differences in *LTL* aroma between grazing and housed feeding goats. Among them, only ethyl hexanoate was detected in both feeding groups, contributing sweet, pineapple-like, and fruity notes characteristic of cooked meat and favored by consumers [22]. In contrast, (Z)-4-heptenal, ethyl decanoate, ethyl octanoate and ethyl acetate were specific to the grazing group. These contributed creamy, milky, sweet, and fruity aromas such as apple, banana, grape, and pineapple, indicating that fruity and dairy were the dominant aroma profiles of Arbas cashmere goat meat and that their variation was closely associated with the levels of esters and aldehydes presented in the muscle. These findings are consistent with the results reported by Yang et al. [11] in Sunit sheep, where the housed feeding group showed significantly lower levels of desirable compounds, including pentanal, heptanal, octanal, and 1-octen-3-ol. As esters are mainly formed by the interaction of alcohols and free fatty acids produced through lipid oxidation [23], and aldehydes are generated from the oxidation of polyunsaturated fatty acids [24], we further analyzed the lipidomic profiles of *LTL* muscles under different feeding conditions. This allowed us to explore the lipid-related metabolic differences that may underline variations in aroma composition.

The change in feeding systems led to marked changes in the lipid composition of the *LTL* muscle in Arbas cashmere goats. IMF plays an important role in the formation of meat flavor [25] that was significantly higher in goats raised under housed feeding conditions. IMF is primarily composed of TG, TC, and PLIP, and serves as an essential reservoir of flavor precursors in meat [26]. While TC levels did not differ between the two feeding systems, TG and PLIP contents were significantly elevated in the housed feeding group, suggesting enhanced lipid accumulation that may influence the synthesis and transformation of lipid-derived volatile compounds. Previous studies have established a strong link between lipid metabolism and meat flavor development [27]. Comparative analyses in sheep have also shown that grazing and housed feeding result in distinct muscle lipid profiles [10]. In particular, elevated levels of PE and PC are associated with increased free fatty acid content in muscle tissue [28], and their breakdown during meat processing can yield a variety of VOCs, contributing to a richer aroma profile [29]. In our study, TG, PE, PC, and CL were the predominant lipid classes in the *LTL* muscle. Among these, TG showed the highest abundance, especially those enriched in long-chain saturated fatty acids, which are prone to rapid deposition in lipid droplets [30]. For instance, TG (16:0/18:0) has been identified as a key driver of IMF accumulation [31], potentially explaining the higher IMF levels observed in housed feeding goats. However, the impact of certain TG species on meat aroma may not always align with IMF deposition trends. In this study, the total abundance of PE and PC was lower in housed feeding goats compared to grazing goats, which corresponded with a decrease in key flavor compounds. This aligns with previous findings that glycerophospholipids such as PE and PC play a pivotal role in the formation of aroma compounds during meat processing [32]. Glycerophospholipids were not only the dominant lipid class in Arbas cashmere goat muscle but also the most significantly affected by the feeding system, indicating their potential role as key modulators of meat flavor. Based on these findings, we hypothesize that differentially expressed lipid molecules, particularly within the glycerophospholipid class may influence the biosynthesis and transformation of aroma compounds in Arbas cashmere goat meat, ultimately contributing to its sensory quality. However, the direct causal relationship between these specific lipid precursors and volatile compounds remains to be experimentally verified.

The present study identified 37 differentially expressed lipid molecules, with functional enrichment analyses indicating their involvement in glycerophospholipid metabolism, arachidonic acid metabolism, and α-linolenic acid metabolism. Among these, only PC (15:0/22:6) was significantly enriched, suggesting its potential as a key lipid species in regulating meat flavor. To explore its regulatory role, we further analyzed lipids significantly associated with PC (15:0/22:6). Lipid species such as PG (16:0/20:5), PC (12:1e/22:5), PE (16:1/20:5), PE (22:5/18:2), PE (16:0/20:5), CL (20:5/16:0/18:0/20:4), CL (20:5/16:0/20:0/20:4), CL (20:5/16:1/18:1/20:4), and PS (20:5/20:4) exhibited positive correlations with PC (15:0/22:6), whereas AcCa (20:1), AcCa (20:0), PE (18:0p/22:5), CL (17:0/18:0/18:2/22:0), AcCa (22:1), CL (23:0/18:2/18:1/20:1), PC (10:0/22:3), CL (23:0/18:1/18:2/20:0), Hex1Cer(t16:1/18:1) and TG (4:0/12:3/18:1) showed negative correlations. Further correlation analysis revealed that PC (15:0/22:6) was negatively associated with IMF, TG, and PLIP levels in muscle. Notably, lipids positively correlated with PC (15:0/22:6) also exhibited strong negative correlations with these fat-related traits, whereas lipids negatively correlated with it, particularly TGs, AcCa, and Hex1Cer showed significant positive associations with IMF, TG, and PLIP accumulation. These findings suggest that lipid accumulation in Arbas cashmere goat muscle is more susceptible to modulation by TG and AcCa species, while PC (15:0/22:6) and its positively associated glycerophospholipids (PE, CL, PC, PS, PG) may play a more pivotal role in aroma formation. PC is a critical phospholipid for lipoprotein assembly and secretion [33], and is the most abundant glycerophospholipid in skeletal muscle. It is highly susceptible to degradation during thermal processing, generating a wide range of volatile compounds and thus playing a central role in meat aroma development [34]. Its involvement in glycerophospholipid metabolism supports lipid biosynthesis and the generation of VOCs in meat products [35]. Notably, docosahexaenoic acid (DHA, C22:6) as a polyunsaturated fatty acid within PC, has been positively associated with meat quality and flavor acceptability [36]. DHA is an established aroma precursor that undergoes oxidation to form key aldehydes contributing to the sensory profile of meat [37]. In contrast, C15:0 as a saturated fatty acid component of PC (15:0/22:6), enhances membrane rigidity and protects muscle cells from oxidative damage [38], further reinforcing the functional significance of this phospholipid in aroma development. To validate the involvement of PC (15:0/22:6) in aroma regulation, we examined its correlation with six key volatile aroma compounds identified in this study: (Z)-4-heptenal, ethyl hexanoate, ethyl decanoate, ethyl octanoate, ethyl acetate, and ethyl 3-methylbutyrate. PC (15:0/22:6) and its positively associated lipids showed strong positive correlations with all six aroma compounds, indicating that downregulation of this molecule in housed feeding goats may contribute to diminished aroma. In contrast, AcCa species, which were negatively correlated with PC (15:0/22:6), exhibited negative associations with the same key aroma compounds. This is consistent with reports by Tonazzi et al. [39] and Zhang et al. [40], who found that AcCa, released via carnitine palmitoyltransferase II during fatty acid β-oxidation, may promote lipid oxidation and reduce aromatic integrity. Evidence across ruminant species indicates that the odd-chain fatty acid C15:0 was a reliable marker of the feeding system. In lamb and beef cattle research, C15:0 distinguished pasture-fed, semi-housed and fully housed production [41, 42]. Dairy studies showed the same system: compared with grass-fed cows, total mixed ration feeding lowers milk C15:0 by 50% [43] and higher grazing intensity increased C15:0 alongside other nutritionally important unsaturated fatty acids, allowing clear discrimination among feeding systems [44]. These findings support the hypothesis that the shift from grazing to housed feeding altered PC (15:0/22:6) accumulation in goat *LTL* muscle, and this change is causally linked to the reduction in characteristic meat aroma.

These findings highlight the important role of lipid molecules in regulating meat aroma and quality in goats. Further investigation is needed to elucidate the mechanisms by which the phospholipid PC (15:0/22:6) influences the formation of VOCs in muscle. In addition, ongoing research may support the development of precision nutrition strategies to improve meat quality in intensive goat production patterns. However, this study had certain limitations. Due to the relatively small sample size, the findings should be further validated and extended in larger samples and confirmed using more advanced sequencing technologies.

## Conclusion

This study compared the effects of grazing and housed feeding regimes on the aroma profiles of the *LTL* muscle in Arbas cashmere goats and employed integrated foodomics and lipidomics approaches to reveal significant differences in aroma characteristics and lipid composition between the two feeding systems. The aroma of the *LTL* muscle in Arbas cashmere goats was predominantly characterized by fruity and milky notes. Notably, key aroma compounds, such as 4-heptenal, ethyl hexanoate, ethyl decanoate, ethyl octanoate, and ethyl acetate, were found to be more abundant in the grazing group, while their levels were lower in the housed feeding group. Lipidomics analysis identified that PC (15:0/22:6) plays a vital role in glycerophospholipid metabolism, arachidonic acid metabolism, and α-linolenic acid metabolism pathways, indicating its crucial role in the synthesis of aroma-related compounds. Housed feeding significantly promoted intramuscular fat deposition but also led to a reduction in PC (15:0/22:6), thereby decreasing the overall abundance of key aroma substances. These findings demonstrate that shifts in feeding systems can suppress the accumulation of lipid-derived aroma precursors in muscle, thereby diminishing meat flavor quality. This highlights a potential drawback of intensive production systems. Future research should focus on enhancing the deposition of functional lipid precursors to improve the flavor quality of goat meat, building upon the specific metabolic traits observed in Arbas Cashmere goats under natural pasture grazing versus house-feeding regimens, to support the genetic and nutritional improvement of local goat breeds in China.

## Supplementary Information

Additional file 1: Table S1. Ingredients and nutritional level of diets (dry matter basis, %). Table S2. Electronic nose aroma characteristics of Arbas cashmere goat meat under different feeding patterns. Table S3. Qualitative and quantitative results of volatile compounds in Arbas cashmere goat meat under different feeding patterns. Table S4. Major components with ROAV ≥1 of Arbas cashmere goat meat under different feeding patterns. Table S5. Differential VOCs with ROAV ≥ 0.1 of Arbas cashmere goat meat. Fig. S1. Aroma compounds of the *LTL* muscle in Arbas cashmere goats between GRA and HFD group. Fig. S2. Differences in the composition of aroma compounds in the *LTL* muscle of Arbas cashmere goats under different feeding patterns. Fig. S3. Fatty acid levels of the *LTL* muscle in Arbas cashmere goats.

## Data Availability

All data generated or analysed during this study are included in this published article and its supplementary information files.

## Abbreviations

AcCa: Acylcarnitines
AEA: Anandamide
C18:2n-6: Linoleic acid
C18:3n-3: α-Linolenic acid
Cer: Ceramides
CerP: Ceramide phosphates
CL: Cardiolipins
CmE: Ceramide methyl ester
Co: Coenzyme A derivatives
DG: Diglycerides
dMePE: Dimethylphosphatidylethanolamines
GM: Glycosphingolipids
GL: Glycerolipids
GP: Glycerophospholipids
GRA: Grazing
FA: Fatty acids
Hex1Cer: Hexosylceramides
HFD: Housed feeding
HS–SPME–GC–MS: Headspace Solid-Phase Microextraction-Gas Chromatography-Mass Spectrometry
IMF: Intramuscular fat
KEGG: Kyoto Encyclopedia of Genes and Genomes
LDA: Linear Discriminant Analysis
LPC: Lysophosphatidylcholines
*LTL*: *Longissimus thoracis et lumborum*
MePC: Methylphosphatidylcholines
MG: Monoglycerides
MGDG: Monogalactosyldiacylglycerols
MTBE: Methyl tert-butyl ether
PC: Phosphatidylcholines
PE: Phosphatidylethanolamines
PG: Phosphatidylglycerols
phSM: Phosphosphingomyelins
PI: Phosphoinositides
PS: Phosphatidylserines
SP: Sphingolipids
SPH: Sphingoid bases
SPHP: Sphingosine phospholipids
SM: Sphingomyelins
ST: Steryl ester
StE: Stigmasteryl ester
STs: Sterols
TC: Total cholesterol
TG: Triglyceride
OAHFA: (O-acyl)-1-hydroxy fatty acids
PCA: Principal component analysis
PLIP: Phospholipid
PUFAs: Polyunsaturated fatty acids
ROAV: Relative odor activity value
ROAV_A_: ROAV values
VIP: Variable importance in projection
VOCs: Volatile organic compounds
WE: Wax esters
ZyE: Zymosterol ester
