# Supplementary material for: Comparative lipidomics and volatile profiling reveal distinct aroma signatures in Arbas cashmere goat meat from grazing and housed feeding systems
Source: J Anim Sci Biotechnol. 2026 Jun 22;17:126. doi: 10.1186/s40104-026-01423-w (PMC13285302; doi:10.1186/s40104-026-01423-w)
Supplement: Supplementary file 1 — Additional file 1: Table S1. Ingredients and nutritional level of diets (dry matter basis, %). Table S2. Electronic nose aroma characteristics of Arbas cashmere goat meat under different feeding patterns. Table S3. Qualitative and quantitative results of volatile compounds in Arbas cashmere goat meat under different feeding patterns. Table S4. Major components with ROAV ≥1 of Arbas cashmere goat meat under different feeding patterns. Table S5. Differential VOCs with ROAV ≥ 0.1 of Arbas cashmere goat meat. Fig. S1. Aroma compounds of the LTL muscle in Arbas cashmere goats between GRA and HFD group. Fig. S2. Differences in the composition of aroma compounds in the LTL muscle of Arbas cashmere goats under different feeding patterns. Fig. S3. Fatty acid levels of the LTL muscle in Arbas cashmere goats. [file 40104_2026_1423_MOESM1_ESM.docx]

**Table S1** Ingredients and nutritional levels of diets (dry matter basis, %)

| **Items** | **Treatments^1^** | |
| --- | --- | --- |
|  | **HFD** | **GRA** |
| Ingredients, % | | |
| Corn Stover | 16.00 | - |
| Alfalfa | 12.00 | - |
| *Caragana microphylla* | 8.00 | - |
| Corn | 38.20 | - |
| Cottonseed meal | 6.00 | - |
| Canola meal | 5.00 | - |
| Wheat bran | 10.00 | - |
| Salt | 0.80 | - |
| Premix (4%)^2^ | 4.00 | - |
| Total | 100 | - |
| Nutrient level, % | | |
| Dry matter | 92.47 | 96.16 |
| Crude protein | 12.43 | 9.39 |
| Ether extract | 1.51 | 2.70 |
| Starch | 23.58 | 2.41 |
| Neutral detergent fiber | 35.80 | 64.41 |
| Acid detergent fiber | 22.23 | 43.53 |
| Ash | 12.11 | 25.55 |
| Calcium | 1.17 | 2.94 |
| Phosphorus | 0.33 | 0.16 |

^1^GRA, grazing system; HFD, housed feeding system

^2^Premix: Vitamin A 4×10^5^ IU/kg, Vitamin D_3_ 1.5×10^5^ IU/kg, Vitamin E 400 mg/kg, Vitamin K 40 mg/kg, Niacin 200 mg/kg, Biotin 0.8 mg/kg, Fe 1,250 mg/kg, Cu 300 mg/kg, Mn 800 mg/kg, Zn 800 mg/kg, I 12.5 mg/kg, Se 20 mg/kg, Co 8 mg/kg

**Table S2** Electronic nose aroma characteristics of Arbas cashmere goat meat under different feeding systems

| **Sensors^2^** | **Treatments^1^** | | **SEM^3^** | ***P* value** |
| --- | --- | --- | --- | --- |
|  | **GRA** | **HFD** |  |  |
| W1C | 0.064 | 0.523 | 0.07 | <0.001 |
| W5S | 14.812 | 3.388 | 1.85 | <0.001 |
| W3C | 0.104 | 0.642 | 0.08 | <0.001 |
| W6S | 1.215 | 1.142 | 0.01 | <0.001 |
| W5C | 0.000 | 0.620 | 0.09 | <0.001 |
| W1S | 9.089 | 3.152 | 0.93 | <0.001 |
| W1W | 22.192 | 8.188 | 2.20 | <0.001 |
| W2S | 9.623 | 3.527 | 0.93 | <0.001 |
| W2W | 7.724 | 3.891 | 0.61 | <0.001 |
| W3S | 1.909 | 1.757 | 0.03 | <0.001 |

^1^GRA, grazing system; HFD, housed feeding system

^2^Sensors: W1C =Aromatic, W5S = Broad range, W3C = Aromatic, W6S = Hydrogen, W5C = Arom-aliph, W1S = Broad-methane, W1W = Sulphur-organic, W2S = Broad-alcohol, W2W = Sulph-chlor, W3S = Methane-aliph

^3^SEM Standard error of the mean

**Table S3** Qualitative and quantitative results of volatile compounds in Arbas cashmere goat meat under different feeding systems

| **Compound** | **CAS Number** | **Treatments^1^ (%)** | |
| --- | --- | --- | --- |
|  |  | **GRA** | **HFD** |
| **Esters** | | | |
| Ethyl acetate | 141-78-6 | 0.007310491 | 0.000555968 |
| Ethyl propanoate | 105-37-3 | 0.001598021 | 1.10523E-05 |
| Ethyl 3-methylbutanoate | 108-64-5 | 0.000187431 | 1.10523E-05 |
| Ethyl pentanoate | 539-82-2 | 0.00047263 | 3.94632E-05 |
| Ethyl hexanoate | 123-66-0 | 0.012026363 | 0.002650595 |
| Hexyl hexanoate | 106-30-9 | 0.001723763 | 0.000292958 |
| Ethyl heptanoate | 106-32-1 | 0.00514588 | 0.000464326 |
| Ethyl octanoate | 623-42-7 | 2.23878E-05 | 0.000110029 |
| Ethyl nonanoate | 123-29-5 | 0.003502719 | 1.10523E-05 |
| Ethyl decanoate | 106-70-7 | 0.000164074 | 0.00028994 |
| Methyl butanoate | 110-38-3 | 0.013783226 | 0.001049011 |
| Methyl hexanoate | 124-06-1 | 0.001794282 | 1.10523E-05 |
| Butyl butanoate | 628-97-7 | 0.001573752 | 1.10523E-05 |
| Ethyl tetradecanoate | 6114-18-7 | 0.000170089 | 1.10523E-05 |
| Ethyl hexadecanoate | 108-64-5 | 0.001598021 | 1.10523E-05 |
| Ethyl (E)-9-octadecenoate | 539-82-2 | 0.000187431 | 1.10523E-05 |
| Butyrolactone | 96-48-0 | 0.00073605 | 0.000852289 |
| **Alcohols** | | | |
| 1-Propanol | 71-23-8 | 0.01717853 | 0.005177279 |
| 1-Propanol, 2-methyl- | 78-83-1 | 0.000421239 | 1.10523E-05 |
| 1-Butanol | 107-98-2 | 0.020493185 | 0.00712353 |
| 1-Penten-3-ol | 71-36-3 | 0.008134101 | 0.005733634 |
| 3-Hexanol | 623-37-0 | 0.000319051 | 0.000267599 |
| 1-Pentanol | 71-41-0 | 0.092412268 | 0.109738658 |
| 1-Hexanol | 589-82-2 | 0.000599985 | 0.000546893 |
| 1-Octen-3-ol | 111-27-3 | 0.060090787 | 0.058375241 |
| Cyclohexanol | 108-93-0 | 2.23878E-05 | 0.000109374 |
| 3-Heptanol | 5989-33-3 | 0.000370351 | 0.00043949 |
| 1-Heptanol | 111-70-6 | 0.040030819 | 0.041811492 |
| 1-Octanol | 111-87-5 | 0.020791816 | 0.021600641 |
| Benzyl alcohol | 143-08-8 | 0.000368837 | 0.000214562 |
| 1-Nonanol | 71-23-8 | 0.01717853 | 0.005177279 |
| **Aldehydes** | | | |
| Butanal | 123-72-8 | 0.001240071 | 0.001078694 |
| 2-Methylbutanal | 96-17-3 | 0.001383701 | 0.00028244 |
| Pentanal | 110-62-3 | 0.071755285 | 0.092526775 |
| Hexanal | 66-25-1 | 0.23330363 | 0.299358234 |
| Heptanal | 111-71-7 | 0.121781269 | 0.122515945 |
| (Z)-4-Heptenal | 6728-31-0 | 0.00326291 | 0.000349944 |
| (E)-2-Heptenal | 18829-55-5 | 0.00052876 | 0.001063995 |
| (E)-2-Octenal | 2548-87-0 | 2.23878E-05 | 3.93724E-05 |
| Octanal | 124-13-0 | 0.02637066 | 0.028722273 |
| Decanal | 112-31-2 | 0.000408717 | 0.000328592 |
| (Z)-6-Nonenal | 2277-19-2 | 2.23878E-05 | 4.25116E-05 |
| **Actones** | | | |
| 2-Butanone | 78-93-3 | 0.004644578 | 0.001358515 |
| 2,3-Butanedione | 431-03-8 | 0.001768978 | 0.001700508 |
| 2,3-Pentanedione | 600-14-6 | 0.015704186 | 0.006793372 |
| 3-Heptanone | 106-35-4 | 0.000582846 | 0.000586019 |
| 2-Heptanone | 110-43-0 | 0.000989817 | 0.001673844 |
| 2-Octanone | 111-13-7 | 0.024476855 | 0.010360508 |
| 6-Methyl-5-hepten-2-one | 110-93-0 | 0.000819011 | 0.000293455 |
| 4-Hydroxy-4-methyl-2-pentanone | 123-42-2 | 0.000532889 | 0.000254276 |
| **Sulfurous compounds** | | | |
| 2-Pentylfuran | 3777-69-3 | 0.004630832 | 0.00885938 |
| Thiazole | 288-47-1 | 2.23878E-05 | 0.000250202 |
| 5-Ethyldihydro-2(3H)-furanone | 695-06-7 | 0.000851042 | 0.001442201 |
| 5-Propyldihydro-2(3H)-furanone | 105-21-5 | 0.000836275 | 0.001017779 |
| Pyrrole | 109-97-7 | 0.000574431 | 0.000215665 |
| 1-(4,5-Dihydro-2-thiazolyl)-ethanone | 29926-41-8 | 0.000718468 | 1.10523E-05 |
| Dimethyl disulfide | 624-92-0 | 2.23878E-05 | 0.000351595 |
| **Aromatics** | | | |
| Benzene | 71-43-2 | 0.000218961 | 0.0012795 |
| Ethylbenzene | 100-41-4 | 0.003128261 | 0.004368145 |
| p-Xylene | 106-42-3 | 0.005017229 | 0.010504848 |
| o-Xylene | 95-47-6 | 0.001574581 | 0.001343885 |
| Styrene | 100-42-5 | 0.00090958 | 0.00077405 |
| 4-Ethylbenzaldehyde | 4748-78-1 | 0.000512744 | 0.000223055 |
| **Haloalkane** | | | |
| Methylene chloride | 75-09-2 | 0.000180185 | 0.000210671 |
| Ethane, 1,1-diethoxy- | 105-57-7 | 0.000223543 | 1.10523E-05 |
| Trichloromethane | 67-66-3 | 0.000575777 | 0.000384518 |
| Ethane, 1,2-dichloro- | 107-06-2 | 9.17078E-05 | 0.000123744 |
| **Acids** | | | |
| Formic acid | 64-18-6 | 2.23878E-05 | 0.000238013 |
| Butanoic acid | 107-92-6 | 2.23878E-05 | 0.000309632 |
| 4-Methylpentanoic acid | 646-07-1 | 5.25489E-05 | 1.10523E-05 |
| **Others** |  |  |  |
| 1-Methoxy-2-propanol | 107-98-2 | 0.020493185 | 0.00712353 |
| cis-α,α,5-Trimethyl-2-vinyltetrahydrofuranmethanol | 5989-33-3 | 0.000370351 | 0.00043949 |

^1^GRA, grazing system; HFD, housed feeding system

**Table S4** Major components with ROAV ≥ 1 of Arbas cashmere goat meat under different feeding systems

| **Code** | **Aroma compound** | **Odor threshold** | **Odor description** | **Treatments^1^ (ROAV)** | |
| --- | --- | --- | --- | --- | --- |
|  |  |  |  | **GRA** | **HFD** |
| **Esters** | | | | | |
| 1 | Ethyl Acetate | 5.0 | Pineapple, Sweet, Anise, Fruity, Balsam, Weedy, Green, Characteristic Ether-Like Odor Reminiscent of Pineapple, Fragrant Odor, Fruity witha brandy note, Aromatic, Brandy, Grape, Solvent, Eedy, Reen | 1.513 | 0.099 |
| 2 | Ethyl 3-methylbutanoate | 0.01 | Apple, Pineapple, Tutti Frutti, Sweet, Fruity, Sour, Grape, Isovaleric | 19.395 | 0.980 |
| 3 | Ethyl hexanoate | 0.3 | Pineapple, Apple Peel, Waxy, Banana, Fruit, Sweet, Fruity, Green, Apple Peel, Brandy, Fruit Gum, Overripe Fruit, Aniseed, Apple, Unripe | 41.483 | 7.831 |
| 4 | Ethyl octanoate | 5.0 | Apricot, Fat, Wine, Waxy, Banana, Brandy, Fruit, Sweet, Fruity, Pear, Apricot, Brandy, Fat, Floral, Pineapple, Apple | 1.065 | 0.082 |
| 5 | Ethyl decanoate | 8.0 | Apple, Brandy, Waxy, Grape, Oily, Sweet, Fruity, Pear, Brandy, Grape, Pear, Caprylic, Solvent | 1.783 | 0.116 |
| **Alcohols** | | | | | |
| 1 | 1-Hexanol | 10.0 | Oil, Alcoholic, Ethereal, Resin, Fusel, Sweet, Fruity, Green, Characteristic, Sweet Alcohol, Pleasant, Banana, Herb, Fatty, Floral, Grassy | 6.218 | 5.174 |
| 2 | 1-Octen-3-ol | 1.0 | Oily, Mushroom, Green, Cucumber, Floral, Fatty, Fruity, Grass, Sweet, Balsamic | 100.000 | 100.000 |
| 3 | 1-Heptanol | 3.0 | Leafy, Coconut, Herbal, Peony, Strawberry, Sweet, Woody, Violet, Green, Fragrant, Faint, Aromatic, Fatty, Mushroom | 13.808 | 12.354 |
| **Aldehyde** | | | | | |
| 1 | 2-Methylbutanal | 1.0 | Coffee, Cocoa, Nutty, Almond, Fermented, Hazelnut, Malt, Burnt, Estery Apple, Fruity, Green Grass, Sour | 1.43185 | 0.250 |
| 2 | Pentanal | 12.0 | Bready, Fermented, Berry, Malt, Fruity, Nutty, Acrid, Almond, Bitter, Oil, Aldehyde, Banana, Grass, Lower Aldehyde, Oily, Green, Beany, Berry, Coffee, Green, Fatty | 6.188 | 6.835 |
| 3 | Hexanal | 4.0 | Leafy, Tallow, Fresh, Fatty, Fruity, Aldehydic, Green, Characteristic Fruity Odor (On Dilution), Strong, Green Grass Odor, Aldehyde Odor, Apple, Fresh, Oil, Leaves, Vinous, Grassy | 60.356 | 66.337 |
| 4 | Heptanal | 3.0 | Citrus, Herbal, Fresh, Wine-Lee, Fatty, Aldehydic, Green, Penetrating Fruity Odor, Citrus, Nut, Heavy, Oily, Planty Green, Putty, Milky | 42.006 | 36.199 |
| 5 | (Z)-4-Heptenal | 0.4 | Cream, Biscuit, Milky, Oily, Fatty, Creamy, Dairy, Green, Sweet | 8.441 | 0.775 |
| 6 | Octanal | 1.4 | Lemon, Citrus, Soap, Orange Peel, Fat, Waxy, Fatty, Aldehydic, Green, Strong, Fruity Odor, Fatty, Honey Odor On Dilution, Citrus-Like On Dilution, Aldehyde, Fruity, Fresh, Citrus-Like | 19.492 | 18.185 |
| 7 | (Z)-6-Nonenal | 0.005 | Cantaloupe, Green, Melon, Cucumber, Green, Melon Rind | 4.633 | 7.536 |
| **Others** | | | | | |
| 1 | 2-Pentylfuran | 6.0 | Butter, Vegetable, Beany, Fruity, Green, Greenbean, Floral, Sweet, Buttery, Caramel, Roasted, Popcorn | 0.799 | 1.308 |

^1^GRA, grazing system; HFD, housed feeding system

**Table S5** Differential VOCs with ROAV ≥ 0.1 of Arbas cashmere goat meat

| **Code** | **Aroma compound** | **Odor threshold** | **Odor description** | **Treatments^1^ (ROAV)** | |
| --- | --- | --- | --- | --- | --- |
|  |  |  |  | **GRA** | **HFD** |
| **Esters** | | | | | |
| 1 | Ethyl acetate | 5.0 | Pineapple, Sweet, Anise, Fruity, Balsam, Weedy, Green, Characteristic Ether-Like Odor Reminiscent of Pineapple, Fragrant Odor, Fruity With A Brandy Note, Aromatic, Brandy, Grape, Solvent, Eedy, Reen | 1.513 | 0.099 |
| 2 | Ethyl propanoate | 9.0 | Pineapple, Grape, Sweet, Rum, Fruity, Juicy, Butter-Like, Apple, Strawberry | 0.184 | 0.001 |
| 3 | Ethyl 3-methylbutanoate | 0.01 | Apple, Pineapple, Tutti Frutti, Sweet, Fruity, Sour, Grape, Isovaleric | 19.395 | 0.980 |
| 4 | Ethyl pentanoate | 1.5 | Yeast, Apple, Pineapple, Sweet, Fruity, Tropical, Green, Apple, Dry Fish, Herb, Nut | 0.326 | 0.023 |
| 5 | Ethyl hexanoate | 0.3 | Pineapple, Apple Peel, Waxy, Banana, Fruit, Sweet, Fruity, Green, Apple Peel, Brandy, Fruit Gum, Overripe Fruit, Aniseed, Apple, Unripe | 41.483 | 7.831 |
| 6 | Ethyl heptanoate | 2.0 | Pineapple, Wine, Fruit, Plum, Fruity, Melon, Rum, Cognac, Brandy | 0.892 | 0.130 |
| 7 | Ethyl octanoate | 5.0 | Apricot, Fat, Wine, Waxy, Banana, Brandy, Fruit, Sweet, Fruity, Pear, Apricot, Brandy, Fat, Floral, Pineapple, Apple | 1.065 | 0.082 |
| 8 | Ethyl decanoate | 8.0 | Apple, Brandy, Waxy, Grape, Oily, Sweet, Fruity, Pear, Brandy, Grape, Pear, Caprylic, Solvent | 1.783 | 0.116 |
| **Alcohols** | | | | | |
| 1 | 1-Penten-3-ol | 400.0 | Butter, Pungent, Tropical, Horseradish, Green, Vegetable, Bitter, Fruity, Fish, Oxidized, Wet Earth, Burnt, Grass, Slightly Meaty, Mushroom | 0.158 | 0.062 |
| 2 | Benzyl alcohol | 1.2 | Berry, Balsamic, Floral, Walnut, Sweet, Cherry, Phenolic, Grapefruit, Faint Aromatic Odor, Boiled Cherries, Moss, Roasted Bread, Rose | 0.384 | 0.107 |
| **Aldehyde** | | | | | |
| 1 | (Z)-4-Heptenal | 0.4 | Cream, Biscuit, Milky, Oily, Fatty, Creamy, Dairy, Green, Sweet | 8.441 | 0.775 |
| **Actone** | | | | | |
| 1 | 2,3-Pentanedione | 20.0 | Butter, Cream, Caramel, Sweet, Pungent, Creamy, Nutty, Cheese, Buttery, Almond, Burnt, Burnt Butter, Butterscotch, Diacetyl, Estery Apple, Fruity, Grain, Malt, Oily Buttery | 0.813 | 0.301 |
| **Others** | | | | | |
| 1 | Dimethyl disulfide | 3.0 | Citrus, Cabbage, Sulfurous, Putrid, Wine_Like, Onion, Vegetable, Fatty, Floral, Earthy, Woody, Fruity, Herbaceous, Nutty, Meaty, Spicy, Green, Garlic-Like, Sulfurous, Diffuse, Intense Onion Odor, Cabbage, Garlic, Onion, Musty, Cardboard, Sulphur, Fishy, Oniony | 0.008 | 0.104 |
| 2 | p-Xylene | 50.0 | Sweet, Aromatic Odor, Characteristic Odor | 0.104 | 0.186 |

^1^GRA, grazing system; HFD, housed feeding system

**
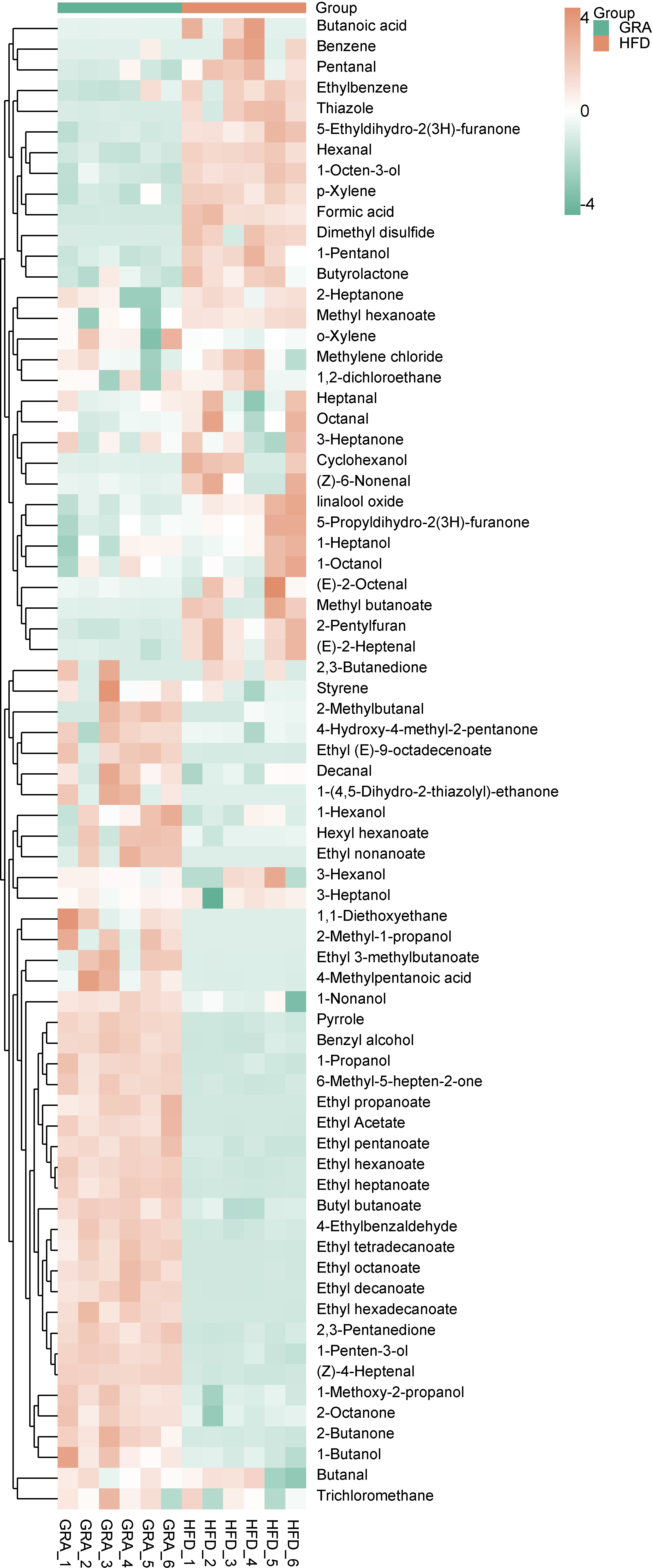
**

**Fig. S1** Aroma compounds of the *LTL* muscle in Arbas cashmere goats between GRA and HFD group identified Heat map analysis of 72 differential aroma compounds


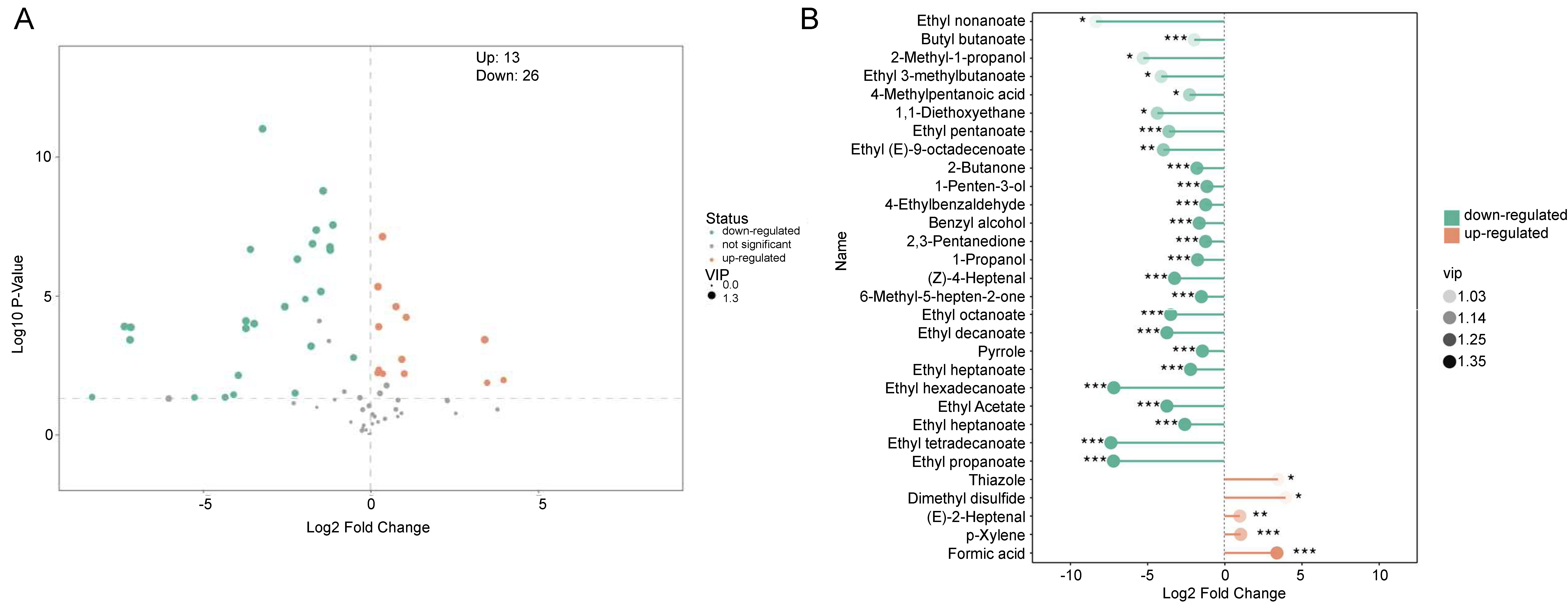


**Fig. S2** Differences in the composition of aroma compounds in the *LTL* muscle of Arbas cashmere goats under different feeding patterns. **A** Volcano plot difference analysis screening criteria: VIP > 1 and *P* < 0.05. **B** Differential aroma compound matchstick graph analysis. Orange indicates positive correlation, and green indicates negative correlation. ^*^0.01 < *P* < 0.05, ^**^0.001 < *P* < 0.01, ^***^*P* < 0.001

**
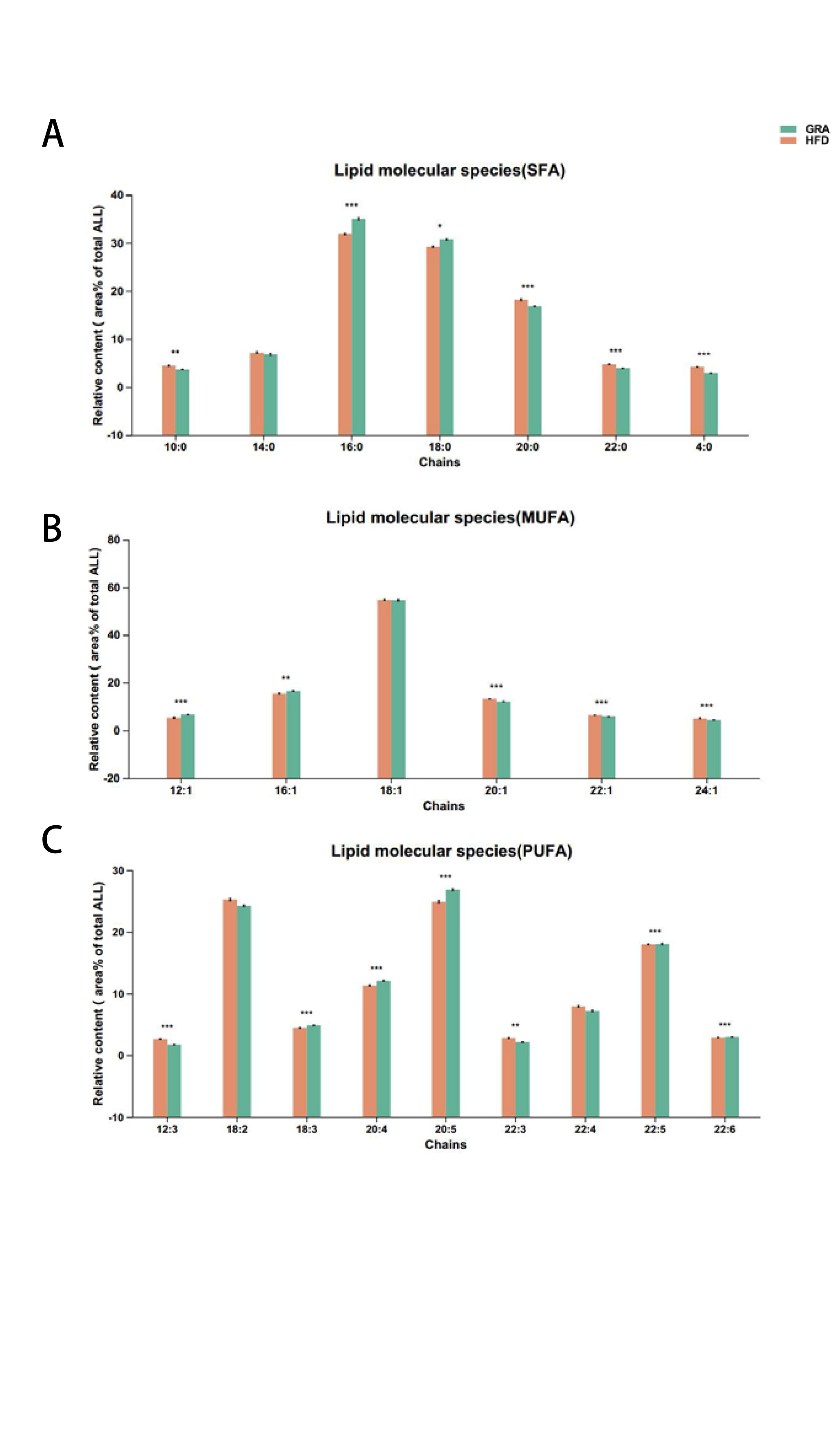
**

**Fig. S3** Fatty acid levels of the *LTL* muscle in Arbas cashmere goats. The content of (**A**) saturated fatty acids (SFA), (**B**) monounsaturated fatty acids (MUFA), and (**C**) polyunsaturated fatty acids (PUFA) in the *LTL* muscle of Arbas cashmere goats under different feeding patterns
